# Effects of dietary supplementation of *Escherichia coli* 6-phytase levels on growth performance, nutrient digestibility, and blood inositol in weanling piglets

**DOI:** 10.5713/ab.25.0186

**Published:** 2025-08-12

**Authors:** Jong Wan Park, Sang Sik Lee, Abdolreza Hosseindoust, Jun Young Mun, Sang Hun Ha, Habeeb Tajudeen, Priscilla Neves Silvestre, So Dam Choi, Seon Ah Park, Santosh Laxman Ingale, Anushka Lokhande, Jin Soo Kim

**Affiliations:** 1 Department of Animal Industry Convergence, Kangwon National University, Chuncheon, Korea; 2 Advanced Enzyme Technologies Ltd., Thane, India

**Keywords:** Crude Protein, Gross Energy, Phosphorus, Phytase, Phytate, Weanling Pig

## Abstract

**Objective:**

This study aimed to investigate the effect of different levels of phytase supplementation on growth performance parameters, nutrient digestibility, amino acid digestibility, and blood inositol concentration in weanling piglets that fed corn-soybean meal diet.

**Methods:**

Weanling piglets were allocated to one of the five feeding treatments: control, a corn-soybean meal diet; dietary supplementation of 500 FTU/kg phytase; dietary supplementation of 750 FTU/kg phytase; dietary supplementation of 1,000 FTU/kg phytase; dietary supplementation of 1,500 FTU/kg phytase. The experiment had two feeding phases (phase 1, d 1 to 21; phase 2, d 22 to 42) to clarify effects of dietary phytase supplementation.

**Results:**

The final body weight, average daily gain, and gain-to-feed ratio in the whole experimental period were increased linearly in response to elevated levels of dietary phytase supplementation (p<0.01). There was no significant difference in average daily feed intake in the whole experimental period. In phase 1, there was a linear increase in apparent total tract digestibility of crude protein (CP), gross energy, and calcium as the level of dietary phytase supplementation increases. Phosphorus digestibility tended to be linearly increased (p = 0.056). In phase 2, linear increases were observed (p<0.01) in calcium and phosphorus digestibility. Additionally, CP digestibility was linearly increased (p<0.05). Dry matter digestibility showed a quadratic effect (p<0.05). The digestibility of arginine, leucine, lysine, tryptophan, alanine, and phenylalanine and cysteine showed a linear increase (p<0.01). There was a linear increase (p<0.01) in blood myo-inositol levels in phase 2.

**Conclusion:**

Dietary phytase supplementation at levels greater than 1,000 FTU/kg significantly improved growth performance, nutrient digestibility, and blood myo-inositol concentrations in weanling pigs. However, further studies are necessary to investigate the effects of super dosing and to determine the optimal phytase dosage.

## INTRODUCTION

Phytate is the primary storage form of phosphorus (P) in plant-based feed ingredients and is indigestible for monogastric animals due to their lack of endogenous phytase enzymes [1,2]. P is an essential yet costly mineral that plays a critical role in cellular metabolism, protein synthesis, skeletal development in piglets, and the regulation of metabolic enzymes [3,4]. Additionally, the complex structure of phytate allows it to chelate and reduce the bioavailability of other minerals, including calcium (Ca), potassium, iron, zinc, and magnesium [1,5]. It can also encapsulate amino acids (AA) and other nutrients, further hindering their absorption [6]. Furthermore, excessive excretion of phytic acid from livestock manure contributes to environmental pollution by increasing P runoff into water systems, which can lead to eutrophication [5]. In swine production, weanling pigs are particularly susceptible to digestive challenges due to the stress associated with weaning and the degradation of phytate is essential to enhance nutrient release and improve digestibility.

To increase the bioavailability of Ca and P, exogenous phytase is commonly added to the diets of weanling piglets [7–9]. 3-Phytases and 6-phytases are the primary types of commercial phytases utilized in animal nutrition. Generally, 3-phytases, commonly derived from *Aspergillus* species [10] hydrolyze phytate by targeting the phosphate group attached to the third carbon of the inositol ring [11,12]. These enzymes are more widely used due to their lower production costs. Conversely, 6-phytases, typically originating from *Escherichia coli*, target the phosphate group at the sixth carbon of phytate. This specificity often results in more efficient dephosphorylation of the inositol structure compared to 3-phytases [10,13]. A key advantage of 6-phytases lies in their optimal activity under neutral pH conditions, enhancing their efficacy in the small intestine [14]. Myo-inositol released from phytic acid degradation can support the growth and development of animals by acting as an essential nutrient [5,15,16]. Although extensive research has been conducted to address growth retardation caused by phytate-derived P deficiency, studies evaluating the efficacy of 6-phytase remain ongoing. *Pichia pastoris* is a widely used expression system for recombinant protein production, including industrial enzymes such as phytase [8]. This yeast offers several advantages, including cost-effective fermentation, and the ability to perform post-translational modifications [14]. Consequently, this study aims to determine the optimal dosage of exogenous *E. coli* 6-phytase produced by controlled fermentation of *Pichia pastoris* in weanling pigs.

## MATERIALS AND METHODS

### 6-Phytase preparation

The *E. coli* 6-phytase used in this study was produced through controlled fermentation (Advanced Enzyme Technologies). The enzyme is presented as cream to off-white colored granules with an activity level of 10,000,000 FTU/kg and has been found to remain heat-stable at temperatures up to 85°C.

### Animals, diet, treatment, housing

The experiment was conducted in two distinct phases (Phase 1, d 1 to 21; phase 2, d 22 to 42). This trial was conducted at the commercial swine farm in Haman, South Korea, from June until July 2023. Four hundred cross-bred (Duroc× Landrace×Yorkshire) newly weanling 21-day-old weanling piglet’s initial body weight (BW) was 6.42±0.01 kg. Those were allotted to one of five dietary treatments: control (CNT), a corn-soybean meal basal diet that met or surpassed the requirements of nutrients referred to the NRC [17] guidelines; CNT with 500 FTU/kg; CNT with 750 FTU/kg; CNT with 1,000 FTU/kg; CNT with 1,500 FTU/kg. The levels of phytase supplementation (0, 500, 750, 1,000, and 1,500 FTU/kg) were selected to represent a stepwise increase from standard commercial doses to higher-than-conventional levels. This range was designed to evaluate the dose-response relationship within a physiologically relevant window, while avoiding levels typically considered as super-dosing. The incremental spacing also allowed for the use of orthogonal polynomial contrasts to assess linear and non-linear trends in growth and nutrient utilization parameters. Each treatment had ten replicated pens which assigned eight weanling piglets per pen for group housed. The sex ratio of weanling piglets in each pen is 4:4 (4 castrated male and 4 female) and applied to every pen. The experiment space consists of 50 pens (2.28×1.85 = 4.2 m^2^) with a partially slatted concrete floor. The environment was controlled by forced ventilation, automated fan-assisted central heating, and artificial lighting to provide a least of 12 hours light in a day. Each pen was equipped with two feeding points-type nipple drinker and one hopper-type feeder. Through the whole experimental period, both water and feed were offered *ad libitum* to weanling piglets and the pens were cleaned daily. The formula and chemical composition of the diets on an as-fed basis are presented in Table 1.

### Growth performance

The BW and cumulative feed intake were measured on the final day of each phase. Through the whole experimental period, growth performance was evaluated at the initial and final days of both phases by calculating data related to average daily weight gain (ADG), average daily feed intake (ADFI), BW, and gain-to-feed ratio (G:F). The ADG was determined by calculation of dividing the weight gain of each weanling piglet through the whole phase of the experimental period by 21 days (d 1 to 21, d 22 to 42). The ADFI was determined by calculation of dividing the cumulative feed intake of each experiment phase by 21 days (d 1 to 21, d 22 to 42). The G:F was calculated by dividing the ADG by the ADFI. Throughout the whole experimental period, mortality was monitored, and no mortality was observed.

### Sample collection and chemical analysis

The weanling piglets were fed a 2.5 g/kg chromium oxide (Cr_2_O_3_) contained feed for seven days (d 15 to 21, d 36 to 42) of each experimental period to analyze the nutrient apparent total tract digestibility (ATTD). On days 20 to 21 and 41 to 42, the fecal samples of the weanling piglets were collected. After collecting all fecal samples from each pen, the samples were frozen at −20°C for subsequent assessment. On days 21 and 42, the blood samples for inositol assay were collected by 2 weanling piglets from the anterior vena cava (castrated male and female) of each pen. Randomly selected two weanling piglets (one castrated male and one female) from each pen were anesthetized using pentobarbital sodium at a dose of 200 mg/kg BW and subsequently euthanized through exsanguination at the end of the experiment. Digesta samples collected from the terminal of the ileum were obtained and reserved after being flushed with distilled water and stored at −20°C until AA ileal digestibility was calculated.

### Apparent total tract digestibility of nutrient

The ATTD of dry matter (DM), crude protein (CP), gross energy (GE), ether extract (EE), ash, Ca, and P was calculated using Cr_2_O_3_ concentration as a marker. The frozen sample was defrosted and desiccated in an air-forced oven (model FC-610, Advantage, Toyo Seisakusho) operating at 75°C for 72 hours. At the beginning of the assay, the dried fecal samples were finely ground through a one-millimeter screen (Christy and Norris Hammer Mill). The GE content was measured by a bomb calorimeter (Parr 1261 bomb calorimeter; Parr Instruments). The CP content assessment was performed using the Kjeldahl method AOAC International 990.03 [18]. The EE content was estimated with AOAC International method 2003.6 [18] and a Soxhlet device (Soxtec 2050; FOSS North America) was used for EE extraction. The ash content was analyzed with AOAC International method 942.05 [18]. The Ca and P contents were assessed with AOAC International method 985.01 [18] after the samples were digested by concentrated nitric acid and 70% perchloric acids. The concentration of Ca was measured by a flame atomic absorption spectrometer (Varian AA FS240; Varian). The concentration of P was measured by a spectrophotometer (Jasco V-550; Jasco) as followed by a previous study [19]. Briefly, samples were digested using concentrated nitric acid and 70% perchloric acid. After digestion, a blue chromogenic reaction was induced by adding acid molybdate and Fiske and Subbarow Reducer (Sigma-Aldrich). The absorbance of the resulting solution was then measured at 620 nm using a spectrophotometer. The concentration of chromium was determined by a spectrometer (Jasco V-650; Jasco).

### Ileal amino acid digestibility

The fresh samples were kept in a glass tube with vacuum condition and hydrolyzed by 6 N HCl (including phenol) for 24 hours at 110±2°C. The classification of AA was confirmed by the Waters Ion Exchange High-Performance Liquid Chromatography system and software (ver. 3.05.01, Millennium, Waters) was used to link the chromatograms.

### Plasma myo-inositol

The concentration of myo-inositol was measured in accordance with a previous study [20]. Proteins in plasma samples were precipitated through the addition of acetonitrile, and the samples were subsequently lyophilized prior to derivatization. A two-step derivatization process, involving oxidation followed by salinization, was employed. Deuterated myo-inositol was used as an internal standard, and myo-inositol levels were quantified using an Agilent 5977A gas chromatograph coupled with a mass spectrometer (Agilent Technologies).

### Statistical analysis

The statistical analysis of this experiment was computed by Analysis of Variance through the General Linear model (GLM) contained in SAS software ver. 9.2 (SAS Institute). Orthogonal polynomial contrasts were utilized to calculate linear and quadratic effects of the supplementation of phytase. The experiment was designed as a randomized complete block design with phytase treatment as a fixed effect and block as a random effect. Pen was an experimental unit, blocked by the initial weight of weanling piglets. The probability values of p≤0.05 were treated as the significant difference between the mean values. When phytase showed a significant difference in parameters at p<0.05 and p<0.01, the Tukey test was used to compare the difference between means.

## RESULTS

### Growth performance

The effect of phytase supplementation on growth performance is shown in Table 2. In phase 1, weanling piglets exhibited a linear effect in ADG and G:F (p<0.05) with increasing dietary phytase levels, whereas ADFI remained unchanged. In phase 2, ADG and G:F showed a linear effect (p<0.01) with higher phytase levels whereas ADFI showed a tendency of quadratic effect (p = 0.084). Over the entire study, there was a linear effect (p<0.01) in ADG and G:F whereas no significant differences were noted in ADFI.

### Apparent total tract digestibility of nutrient

The effect of phytase supplementation on ATTD is shown in Table 3. In phase 1, the ATTD of DM and EE showed a tendency toward linear effect (p = 0.054 and 0.082, respectively). Piglets fed diets with increasing phytase concentrations demonstrated a linear effect (p<0.05) in CP and GE ATTD. No difference was shown in ash ATTD. Additionally, weanling piglets fed diets with increasing phytase levels showed a linear effect (p<0.05) in the digestibility of Ca, while P ATTD showed a tendency toward linear effect (p = 0.056). In phase 2, a quadratic effect (p<0.05) on DM ATTD. The ATTD of CP showed a linear effect (p<0.05), while GE ATTD did not show any linear effect but tended towards quadratic effects (p = 0.059). Additionally, no differences were found in EE and ash ATTD, whereas the ATTD of Ca and P showed linear and quadratic effects (p<0.01).

### Ileal amino acid digestibility

The effects of varying phytase supplementation on AA digestibility are detailed in Table 4. For indispensable AA, there was a linear effect (p<0.05) in the digestibility of lysine (Lys), phenylalanine (Phe), and leucine (Leu) with higher phytase levels. The digestibility of arginine (Arg), and tryptophan (Trp) were linearly affected (p<0.05) by supplemental phytase, while no differences were shown for histidine, isoleucine, methionine, and threonine. The digestibility of valine showed a tendency towards linear effect (p = 0.06). For dispensable AA, cysteine (Cys) and alanine (Ala) digestibility was linearly affected (p< 0.05) by supplemental phytase. No significant differences were found in the digestibility of aspartate, glutamine, glycine, proline, serine, and tyrosine.

### Plasma myo-inositol

**Figure 1:**
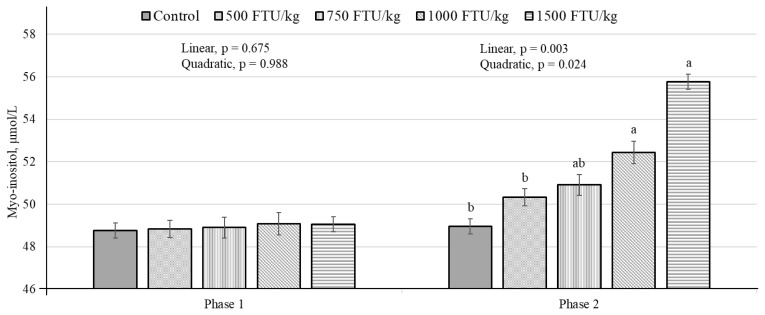
Plasma myo-inositol concentrations (μmol/L) in weaned piglets fed plant-based diets supplemented with 0, 500, 750, 1,000 or 1,500 FTU/kg of phytase on days 21 and 42. ^a,b^ Different letters above bars indicate significant differences among treatments within each sampling day (p<0.05).

In phase 1, there were no significant differences in myo-inositol levels among the dietary treatments (Figure 1). However, in phase 2, a linear increase (p<0.01) in myo-inositol levels was shown with dietary phytase supplementation levels.

## DISCUSSION

In the present study, both ADG and G:F demonstrated a linear improvement with increasing levels of dietary phytase supplementation up to 1,000 or 1,500 FTU/kg. These results correspond with previous studies that indicated a linear effect in ADG and G:F in weanling piglets fed phytase in the diet [4,7,13,21,22]. Moran et al [2] demonstrated that dietary supplementation with 2,500 FTU/kg of 6-phytase resulted in improved growth performance in weanling pigs, exceeding traditional dose levels. This approach, often referred to as “super-dosing,” aims to achieve extra-phosphoric effects in pig diets. Conversely, Gourley et al [23] reported no significant differences in piglet performance during the first three weeks post-weaning when comparing 2,000 to 4,000 FTU/kg of super-dosed 6-phytase levels to 500 to 1,000 FTU/kg conventional doses. While the optimal level of phytase for improving growth performance in weanling pigs has been reported to exceed 500 FTU/kg in previous studies [4,6], its upper threshold for optimal phytase supplementation remains unclear, as it depends on both the specific characteristics of different phytase types. These findings highlight the need for further research to determine the most effective strategy for utilizing 6-phytase in pig nutrition.

In the present study, dietary supplementation of *E. coli* 6-phytase in diets has been associated with improvements in the ATTD of GE, Ca, and P. In aqueous solutions, particularly under acidic conditions, phytic acid carries a negative charge, enabling it to bind with minerals [8,24,25]. Furthermore, phytate can interact with endogenous digestive enzymes, such as α-amylase, sucrase, and maltase, impairing their ability to effectively break down nutrients [1,11,16]. Consequently, the presence of phytate in diets decreased GE, Ca and P ATTD in weaning pigs [26,27]. In agreement, a linear increase in ATTD of DM and GE was observed when higher doses of 250 FTU of phytase were supplemented in corn-soybean meal diets for nursery pigs according to a previous study [28]. However, increasing the *E. coli* phytase dosage up to 4,000 FTU/kg in corn-soybean meal diets did not influence the ATTD of DM or GE in growing pigs [29]. This variation in effectiveness may be attributed to differences in the physiological stage of the pigs, as phytase appears to have greater benefits in younger pigs, likely due to their limited endogenous digestive enzyme activity.

Our current study showed a linear effect in the digestibility of CP and Arg, Leu, Lys, Phe, Tyr, Ala, and Cys. Phytate-protein complexes are less digested by the pepsin and trypsin [30,31]. In particular, Ca is an essential factor for the activation of trypsin; however, its activity is reduced when it forms complexes with phytic acid, leading to a decrease in trypsin activity [26]. Therefore, in our current study, ATTD of CP may be improved by the enhancement of protease bioavailability and degradation of non-specific nature binding with phytate and proteins. Variations in AA ileal digestibility are influenced by the type of AA which composes the protein forming the phytate-protein complex [6,32]. Furthermore, supplementing phytase decreased digesta viscosity and improved nutrient digestibility by breaking down dietary phytate [33,34]. Moreover, the difference in feed ingredient composition can also be a contributing factor in AA digestibility [26]. In agreement, in nursery pigs, the inclusion of 500 FTU/kg of *A. niger* phytase improved ileal AA digestibility in wheat-soybean meal-canola meal-based diets but had no effect in wheat-soybean meal, corn-soybean meal, or barley-pea-canola meal-based diets in a previous study [35]. This suggests that the digesta viscosity and the rate of phytate degradation significantly influences the effectiveness of phytase on ileal AA digestibility, highlighting the need for further studies to determine the optimal phytase inclusion level under varying dietary conditions.

In the present study, while no significant differences in plasma myo-inositol levels were observed due to dietary phytase supplementation during phase 1, a linear effect in these levels was evident in phase 2. The delayed response suggests that a sustained period of phytase activity is required to allow sufficient accumulation of plasma myo-inositol. Blood myo-inositol levels can serve as an indicator of phytase activity levels in monogastric animals and can be considered a nutrient [5,13,36]. Myo-inositol is released from the phytate hydrolysis during digestion and contributes to improved growth performance [2,21,36]. These results indicate that the level of myo-inositol increases with dietary phytase supplementation which could play a role in enhancing growth performance in monogastric animals.

## CONCLUSION

In this study, dietary phytase supplementation in plant-based diets improved ADG, G:F, and the digestibility of Ca, P, and Lys in weanling piglets. Additionally, the increase in blood myo-inositol levels after three weeks of feeding indicates that the phytase efficiently hydrolyzed the phytate and possibly reduced the P excretion. Based on the overall results, phytase supplementation above 1,000 FTU/kg demonstrated significant benefits; however, further research is needed to determine the optimal phytase dosage.

## Figures and Tables

**Table 1:** Ingredients and calculated composition of experimental diets (as-fed diets)

Item	Phase 1	Phase 2
Ingredient composition (%)		
Corn	40.61	55.33
Whey	8.00	-
Fish meal, crude protein 60%	3.00	-
Wheat	6.00	6.00
Soybean meal, crude protein 44%	18.00	24.43
Spray dried plasma protein	4.00	-
Bakery byproduct	5.00	5.00
Sugar	4.00	4.00
Soybean oil	2.50	2.50
Monocalcium phosphate	0.87	1.13
Limestone	1.15	1.09
Salt	0.30	0.30
DL-Methionine (99%)	0.05	0.09
L-Lysine (78%)	0.22	0.41
L-Threonine (99%)	-	0.07
L-Tryptophan (10%)	-	0.04
Vitamin premix^1)^	0.10	0.10
Mineral premix^2)^	0.20	0.20
Lactose	6.00	-
Total	100.00	100.00
Calculated composition (%)		
ME (kcal/kg)	3,400	3,300
Cruce protein	20.00	18.00
SID Lysine	1.35	1.23
SID Methionine	0.39	0.36
SID Methionine+Cystein	0.74	0.68
SID Threonine	0.79	0.73
SID Tryptophan	0.22	0.20
Ca	0.80	0.70
Total P	0.62	0.60

1) Supplied per kilogram of diet: 16,000 IU vitamin A, 3,000 IU vitamin D_3_, 40 IU vitamin E, 5.0 mg vitamin K_3_, 5.0 mg vitamin B_1_, 20 mg vitamin B_2_, 4 mg vitamin B_6_, 0.08 mg vitamin B_12_, 40 mg pantothenic acid, 75 mg niacin, 0.15 mg biotin, 0.65 mg folic acid.

2) Supplied per kilogram of diet: 45 mg Fe, 0.25 mg Co, 50 mg Cu, 15 mg Mn, 25 mg Zn, 0.35 mg I, 0.13 mg Se.

**Table 2:** The effect of phytase dosage levels on growth performance parameters in weanling piglets

Item	Dosage (FTU/kg)					SEM	p-value	Linear	Quadratic

	Control	500	750	1,000	1,500				
Phase 1 (d 0–21)									
ADG (kg)	367.5	381.9	388.9	407.1	392.2	17.58	0.263	0.012	0.315
ADFI (kg)	483.2	481.8	482.6	483.2	486.1	3.93	0.855	0.423	0.429
G:F	0.76	0.79	0.81	0.84	0.81	0.04	0.281	0.017	0.258
Phase 2 (d 22–42)									
ADG (kg)	486.5	497.8	542.5	550.0	548.6	31.82	0.140	0.005	0.647
ADFI (kg)	785.2	807.8	788.2	797.0	775.1	13.01	0.142	0.292	0.084
G:F	0.62	0.62	0.69	0.69	0.71	0.04	0.094	0.003	0.978
Overall (d 0–42)									
ADG (kg)	427.0^b^	439.8^ab^	465.7^ab^	478.6^a^	470.4^a^	14.43	0.003	<0.001	0.266
ADFI (kg)	634.2	644.8	635.4	640.1	630.6	7.13	0.320	0.458	0.171
G:F	0.67^b^	0.68^ab^	0.74^ab^	0.75^a^	0.75^a^	0.02	0.004	<0.001	0.550

a,b Means with different superscripts within a row differ (p<0.05).

SEM, standard error of mean; ADG, average daily weight gain; ADFI, average daily feed intake; G:F, gain to feed ratio.

**Table 3:** The effect of phytase dosage levels on apparent nutrient digestibility in weanling piglets

Item (%)	Dosage (FTU/kg)					SEM	p-value	Linear	Quadratic

	Control	500	750	1,000	1,500				
Phase 1 (d 21)									
DM	80.63	80.75	80.91	81.15	80.99	0.26	0.295	0.054	0.481
CP	81.00	81.15	81.31	81.56	81.44	0.26	0.227	0.032	0.518
GE	80.87	80.88	80.86	81.13	81.35	0.23	0.161	0.025	0.250
EE	70.92	71.08	71.05	70.52	71.16	0.24	0.082	0.867	0.462
Ash	63.85	63.95	64.12	64.24	64.18	0.26	0.564	0.115	0.611
Ca	65.53	65.69	66.03	66.13	66.01	0.25	0.102	0.017	0.231
P	52.44	52.52	52.74	53.04	52.75	0.26	0.187	0.056	0.356
Phase 2 (d 42)									
DM	78.68	78.82	78.93	79.25	78.58	0.26	0.104	0.692	0.044
CP	78.43^b^	78.69^ab^	78.90^ab^	79.28^a^	78.85^ab^	0.27	0.040	0.021	0.092
GE	78.94	78.72	78.76	79.02	79.30	0.24	0.131	0.067	0.059
EE	68.91	68.99	69,27	69.23	69.06	0.27	0.641	0.374	0.274
Ash	61.62	61.77	61.82	62.06	61.82	0.23	0.433	0.186	0.332
Ca	63.15^b^	63.52^ab^	63.88^ab^	64.20^a^	63.81^ab^	0.27	0.006	<0.001	<0.001
P	50.40^b^	50.67^ab^	50.70^ab^	51.13^a^	50.99^ab^	0.23	0.023	<0.001	<0.001

a,b Means with different superscripts within a row differ (p<0.05).

SEM, standard error of the mean; DM, dry matter; CP, crude protein; GE, gross energy; EE, ether extract; Ca, calcium; P, phosphorus.

**Table 4:** The effect of phytase dosage levels on ileal amino acid digestibility in weanling piglets

Item (%)	Dosage (FTU/kg)					SEM	p-value	Linear	Quadratic

	Control	500	750	1,000	1,500				
Indispensable									
Arginine	80.99^b^	81.51^ab^	81.79^ab^	82.28^a^	81.89^ab^	0.34	0.006	0.001	0.077
Histidine	66.67	66.25	66.14	66.62	66.28	0.42	0.619	0.655	0.494
Isoleucine	71.73	71.64	71.16	71.18	71.60	0.43	0.531	0.452	0.185
Leucine	73.19	73.41	73.89	74.26	74.06	0.41	0.065	0.007	0.389
Lysine	81.06	81.39	81.78	82.12	81.83	0.40	0.094	0.015	0.230
Methionine	82.44	82.04	82.45	82.61	82.47	0.42	0.712	0.506	0.809
Phenylalanine	81.94	82.14	82.53	82.82	82.60	0.42	0.219	0.036	0.398
Threonine	64.07	64.29	64.62	64.44	64.49	0.35	0.570	0.207	0.369
Tryptophan	70.01^b^	70.61^ab^	71.05^a^	71.46^a^	70.87^ab^	0.35	0.003	0.002	0.012
Valine	67.34	67.69	67.89	68.21	67.92	0.39	0.262	0.060	0.267
Dispensable									
Alanine	65.97^b^	66.29^ab^	66.52^ab^	67.04^a^	66.76^ab^	0.36	0.045	0.006	0.341
Aspartic acid	72.86	72.82	73.05	72.34	72.74	0.35	0.348	0.358	0.947
Cysteine	59.69	59.11	59.51	59.33	58.72	0.36	0.084	0.037	0.502
Glutamic acid	69.35	69.70	69.39	69.43	69.57	0.35	0.846	0.822	0.936
Glycine	53.89	53.57	53.86	53.16	54.03	0.35	0.123	0.875	0.149
Proline	69.35	69.26	69.59	59.56	69.28	0.37	0.834	0.859	0.451
Serine	73.29	73.78	73.20	73.62	73.40	0.37	0.511	0.944	0.673
Tyrosine	68.52	68.57	68.67	68.38	68.47	0.38	0.952	0.734	0.752

a,b Means with different superscripts within a row differ (p<0.05).

SEM, standard error of the mean.
